# Bio-Psycho-Socio-Spirito-Cultural Factors of Burnout: A Systematic Narrative Review of the Literature

**DOI:** 10.3389/fpsyg.2021.722862

**Published:** 2021-12-01

**Authors:** Ian W. Listopad, Maren M. Michaelsen, Lena Werdecker, Tobias Esch

**Affiliations:** Institute for Integrative Health Care and Health Promotion, Department of Medicine, Faculty of Health, Witten/Herdecke University, Witten, Germany

**Keywords:** burnout, stress, spirituality, work culture, bio-psycho-socio-spirito-cultural model, burnout dimensions, systematic narrative review

## Abstract

**Background:** Burnout is a widespread, multifactorial, and mainly psychological phenomenon. The pathogenesis of burnout is commonly described within the bio-psycho-social model of health and disease. Recent literature suggests that the phenomenon of burnout may be broader so that the three dimensions might not reflect the multifaceted and complex nature of the syndrome. Consequently, this review aims to identify the diversity of factors related to burnout, to define overarching categories based on these, and to clarify whether the bio-psycho-social model adequately describes the pathogenesis of burnout—holistically and sufficiently.

**Method:** Five online databases (PubMed, PubPsych, PsychARTICLES, Psychology and Behavioral Sciences Collection, and Google Scholar) were systematically searched using defined search terms to identify relevant studies. The publication date was set between January 1981 and November 2020. Based on the selected literature, we identified factors related to burnout. We aggregated these factors into a comprehensible list and assigned them to overarching categories. Then, we assigned the factors to the dimensions of an extended model of health and disease.

**Results:** We identified a total of 40 burnout-related factors and 10 overarching categories. Our results show that in addition to biological, psychological, and socio-environmental factors, various factors that can be assigned to a spiritual and work cultural dimension also play an important role in the onset of burnout.

**Conclusion:** An extended bio-psycho-socio-spirito-cultural model is necessary to describe the pathogenesis of burnout. Therefore, future studies should also focus on spiritual and work cultural factors when investigating burnout. Furthermore, these factors should not be neglected in future developments of diagnosis, treatment, and prevention options.

## Introduction

One of the most common psychological symptoms that increasingly affect people is burnout, which is commonly described as a consequence of chronic work-related stress (Melamed et al., 2006; Maslach and Leiter, 2016). Maslach et al. (1996) describe burnout with three subscales: (1) exhaustion, (2) depersonalization/cynicism, and (3) reduced professional efficacy/reduced personal accomplishment. A key aspect of burnout is exhaustion which manifests as feelings of being overextended. Depersonalization/cynicism refers to cynical and negative attitudes toward work and people with whom one interacts (e.g., customers, colleagues). Reduced professional efficacy is related to a lack of performance at work. According to Maslach et al. (1996), burnout is characterized by high levels of emotional exhaustion and depersonalization and low levels of professional efficacy. The Maslach Burnout Inventory (MBI; Maslach et al., 1996) has been developed based on these three subscales. Apart from the MBI, there are also other instruments for measuring burnout [e.g., Shirom-Melamed Burnout Measure (SMBM), Shirom and Melamed, 2006; Oldenburg Burnout Inventory (OLBI), Demerouti et al., 2001; Copenhagen Burnout Inventory (CBI), Kristensen et al., 2005].

The stress model of Folkman and Lazarus (1985) represents an established concept in explaining human stress reactions (Riedl, 2013). Here, stress is not primarily understood as a biological phenomenon but as a complex construct that arises from the interactions between an individual and the demands of a situation and is associated with both negative and positive emotions (Folkman, 2008). Thus, the cognitive appraisal processes of the individual are considered in the stress model. Consequently, the decisive factor for a stress reaction is not primarily the stimulus or the situation in which a stimulus is perceived, but its appraisal by the individual as well as the coping resources (Folkman et al., 1986). However, the triggering of stress reactions does not presuppose the conscious perception of stressors and the possibility of coping with them. Complementing the theory of Folkman et al. (1986), it is also possible in exceptional cases to be (physiologically) stressed without perceiving it and/or without feeling incompetent (Esch et al., 2002; Benson and Stefano, 2005; McEwen, 2007, 2017; Esch and Stefano, 2010; Werdecker and Esch, 2018). In this respect, it would also be possible to experience burnout without noticing that there is, e.g., a coping problem. Thus, stress and its consequences to health is a question of dose, controllability, and possessing the inner resources to cope and manage (Stefano et al., 2005).

In addition to Karasek's (1979) demand-control model and Siegrist (2002) effort-reward imbalance (ERI) model (occupational gratification crisis), which are related to stress and burnout (Violanti et al., 2018; Demerouti and Nachreiner, 2019), there are also other approaches in stress and burnout research. One model to explain the onset of burnout and disengagement is the job demands-resource model (Demerouti et al., 2001). According to the model, variables of work can be divided into demands and resources. An accumulation of demands (e.g., time pressure) can be related to the exhaustion component of burnout. In contrast, job resources (e.g., job control) are associated with work engagement (opposite pole of burnout) through the motivational process of the model. The model was additionally extended by an interaction effect between demands and resources, since existing resources can mitigate the negative effect of demands (Demerouti et al., 2001; González-Romá et al., 2006).

Burnout is a risk that is associated with different stress-related physical (Maslach et al., 2001), psychosomatic (e.g., Melamed et al., 2006), and mental disorders such as depression and anxiety (Koutsimani et al., 2019). Besides consequences for the individual, there are also consequences in occupational health such as reduced work engagement (Schaufeli and Bakker, 2004; Maslach and Leiter, 2008, 2016; Schaufeli et al., 2009), which can be associated with various forms of withdrawal resulting in absenteeism and increased intention to quit (Alarcon, 2011; Kim and Kao, 2014). Thus, burnout not only affects the individual, but incurs costs for employers, the health system, and society in general (Wigert and Agrawal, 2018; Borysenko, 2019; Smith, 2019; Werdecker and Esch, 2020).

### Burnout in the ICD-11 and Current Research

In the ICD-11^1^ of the World Health Organization (WHO), burnout is described on the basis of the three burnout subscales of Maslach et al. (1996) and attributed to chronic stress at work. This means that people develop burnout when they cannot successfully manage work stressors. Furthermore, burnout is classified as an “occupational phenomenon” rather than a medical condition [Werdecker and Esch, 2021; WHO (World Health Organization), 2021].

Given extensive research in the field of stress and stress physiology, burnout and its underlying causes are numerous and complex (e.g., Esch, 2002, 2003; Esch and Stefano, 2010; Werdecker and Esch, 2018, 2021). The symptoms can occur in the case of overload due to accumulation of demands, lack of job resources (Demerouti et al., 2001), as well as inappropriate and insufficient health resources and resistance factors (individual resilience and coherence factors) (Gillespie et al., 2007). In addition, burnout seems to be associated with several factors such as control, fairness, support, reward, coping strategies, personality variables, etc. (Maslach and Leiter, 2008, 2016; Alarcon et al., 2009; Kay-Eccles, 2012; Shin et al., 2014).

Although there are various studies examining burnout, there is a lack of a holistic view—including whether biological, psychological, and socio-environmental factors (based on the established health and disease model) are sufficient to describe the onset of the syndrome.

### A Paradigm Shift in the Context of Burnout?

This hypothesis is based on several strands from the psychological, sociological, and medical literatures. According to various authors, the established bio-psycho-social model by Engel (1977) has limitations (e.g., Suls and Rothman, 2004; Havelka et al., 2009; Babalola et al., 2017; Listopad et al., 2021). The limitations concern, e.g., the isolation between the individual dimensions, lack of consideration for the emotional relationships between patients and professionals, possibility of extension concerning the sociotype, with the subdivisions of the environment into three domains [i.e., individual (intra-personal); relationships (inter-personal), and context (work and leisure environment, etc.)], as well as cultural and spiritual aspects as supplementary dimensions (Sulmasy, 2002; Suls and Rothman, 2004; McGee and Torosian, 2006; Esch, 2008a, 2011, 2019; Havelka et al., 2009; Babalola et al., 2017; Berry et al., 2017; Listopad et al., 2021). There is preliminary evidence that spirituality, meaningfulness, faith, and trust are pain- and stress-reducing and essential within a holistic model of health and disease (Sulmasy, 2002; McGee and Torosian, 2006; Esch, 2008a, 2011, 2019; Saad et al., 2017; Listopad et al., 2021). Likewise, according to Esch (2017), the feeling of “home” (e.g., facets of homeliness, belongingness, and environmental connectedness) is biologically and culturally determined and—analogous to spirituality—should not be missing from a model of health and disease (Esch, 2008a, 2011, 2019; Listopad et al., 2021).

Initial studies indicate that psychological-mental aspects such as inner/intrapersonal resources, e.g., perceived meaningfulness, faith, belief, mindfulness, as well as the feeling of homeliness in one's environment, belongingness, and connectedness are associated with life satisfaction, happiness, work engagement, well-being, resilience, stress, and health maintenance (Esch and Stefano, 2007; Esch, 2008a, 2011, 2019; Caglar, 2011; Daniel, 2014; Van Wingerden and van der Stoep, 2017; Scanlan and Hazelton, 2019). In addition, there are preliminary studies that draw attention to the limitations of the bio-psycho-social model, as some factors (e.g., spirituality, perceived meaningfulness of work, and sense of homeliness) associated with burnout cannot be clearly attributed to Engel's model published in 1977 (Golden et al., 2004; Salmoirago-Blotcher et al., 2016; Carneiro et al., 2019; Esch, 2019; Listopad et al., 2021). However, in this context, the investigation of burnout within the bio-psycho-socio-spirito-cultural model still represents a research gap (Esch, 2019; Listopad et al., 2021) which we aim to address in our study.

### Objectives

The present review aims to identify all relevant burnout factors (variables related to burnout or [chronic] stress) that are discussed in the literature to date. Furthermore, we aim at aggregating these factors into overarching categories and listing and enumerate them to achieve a better understanding. Next, we assign these factors to various dimensions of the (extended) health and disease model.

Consequently, we investigate the following research questions. First, according to the current literature, what are the factors associated with the onset of burnout? Second, to which overarching categories can the identified factors be summarized? Finally, is the bio-psycho-social model sufficient to comprehensively describe the pathogenesis of burnout or is an extension of the model necessary?

## Methods

The present study is based on a systematic narrative approach. First, a systematic literature search with corresponding search codes and defined inclusion and exclusion criteria was performed. Subsequently, based on the underlying objectives of the article, the results were presented using a narrative approach.

### Systematic Search Strategy

To reduce a possible bias in the literature selection and retrieve relevant research studies, a systematic search procedure was conducted (Jahan et al., 2016). For the literature search, databases were selected that contain psychological, medical, as well as social and health science articles. A two-stage search strategy as recommended by various authors was applied (Green et al., 2006; Ressing et al., 2009; Ferrari, 2015). First, the search was conducted in the databases (1) PubMed, (2) PubPsych^2^, (3) PsychARTICLES, (4) Psychology and Behavioral Sciences Collection, and (5) Google Scholar^3^. The search criteria with the various search terms (“burnout,” “determinants,” “variables,” “factors,” and “reasons”) are listed in Table 1.

**Table 1:** Used databases and generic search strings.

**Databases**	**Generic search strings**
PubMed	“Burnout[MeSH] AND (determinants[tiab] OR variables[tiab] OR factors[tiab], OR reasons[tiab] AND (“1981/01/01”[Date - Publication]: “300”[Date - Publication]))”
PubPSYCH	“TI=(“Burnout” (determinants, OR variables, OR factors, OR reasons)) PY>=1981 PY < =2019”
PsychARTICLES	“allintitle: Burnout”
Psychology and Behavioral Sciences Collection	“allintitle: Burnout”
Google Scholar	“allintitle: Burnout AND determinants OR variables”

As already mentioned above, for the PsychARTICLES and Psychology and Behavioral Sciences Collection databases, we decided to conduct an “umbrella” search strategy with the search term “burnout” to attain a high number of eligible studies and not to neglect potentially relevant investigations. This type of search strategy was not possible for PubMed and PubPSYCH due to the high number of hits (almost 20,000 articles in total) and associated processing time involved in such a publication process. Instead, a refined search code was applied here. Secondly, a review of the references was carried out during the full-text screening (“snowball” method). The “snowballing” approach was additionally chosen as it has been recommended by different authors (DePoy and Gitlin, 1993; Green et al., 2006; Karpetis, 2019) as an important technique to fill in possible gaps in the database search.

### Inclusion and Exclusion Criteria

Various empirical quantitative and qualitative studies, reviews, and theoretical articles focusing on burnout were eligible for inclusion in the current review. English and German language studies published between January 1981 and November 2020 have been considered. The year 1981 was chosen due to the development of the MBI (Maslach and Jackson, 1981). The starting date has already been used in another literature review on work stress and burnout by Schaufeli and Peeters (2000). Since burnout is mainly investigated as an “occupational phenomenon” and accordingly listed in the future ICD-11 [WHO (World Health Organization), 2021], the articles included are limited to working adults. In addition, we try to consider comparable populations by focusing on the workforce. Studies that did not refer to burnout (e.g., when burnout was equated with depression and surveyed with a depression inventory) were excluded due to different underlying concepts and potential causes, while identified studies that were related to (chronic) stress at work and therefore associated with burnout [due to the definition in the ICD-11; WHO (World Health Organization), 2021] were included. In addition to cross-sectional and longitudinal studies, reviews (meta-analyses, systematic reviews, and narrative reviews), as well as theoretical articles were reviewed as they also contain relevant results and references on burnout.

### Systematic Analysis Procedure

**Figure 1:**
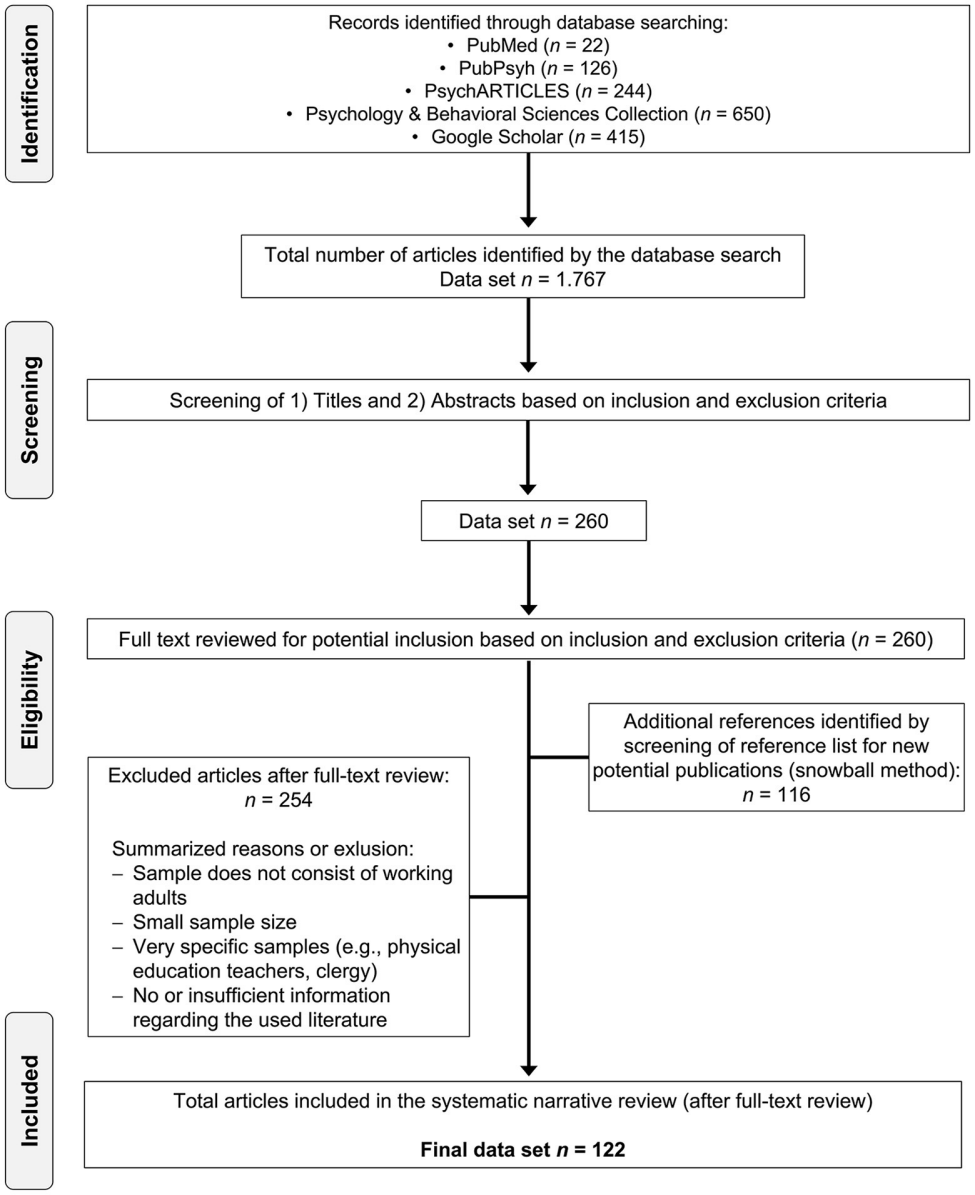
PRISMA flow diagram of the systematic literature selection process.

For each search engine, we documented the search results. After the articles were identified in the corresponding databases, the titles and abstracts were screened. Based on the inclusion and exclusion criteria, 260 articles were eligible (first stage of the search). As mentioned, during the full-text screening, a review of the references was also performed to identify further eligible articles (“snowball technique,” second stage of search). In the second stage of search (reviewing the reference list), 116 additional articles were found. Thus, a total of 376 articles were full-text screened, while 122 articles were finally included in this review (see Figure 1). According to Krause et al. (2015), we used an evidence table (consisting of source/study type, sample, study objective, method, results, general comments, and possible overarching category of identified burnout factors) for a better overview and summary.

## Results

The identified literature was diverse in terms of study design and setting. Although the MBI was predominant, other burnout questionnaires (e.g., SMBM, Shirom and Melamed, 2006; OLBI, Demerouti et al., 2001) were also used. Even though some studies focused on selected occupations and sectors, the results were largely homogeneous with regard to burnout factors. In the following, we will present the findings sorted by factors and their overarching categories. At the end of the chapter, we will assign the factors to the model of health and disease.

### Identified Burnout Factors and Their Superordinate Categories (Research Question 1 and Research Question 2)

The selected studies provide the results according to the current state of the literature. Here, we present the identified factors associated with burnout (research question 1) as well as the corresponding overarching categories (research question 2).

Based on all relevant burnout factors identified through our search, we have aggregated the factors (e.g., appropriate workload/working time) into 10 categories (e.g., working environment) using an evidence table as recommended by Krause et al. (2015) and an inductive category formation (Mayring, 2015). To ensure clarity and structure, as well as contextual relationships, we listed the categories according to the dimensions (biological dimension, psychological dimension, etc.) of the health and disease model; even when factors can be assigned to multiple dimensions. The following categories for the burnout factors were identified: (1) lifestyle, (2) physical and mental health, (3) self-reference, (4) relaxation, (5) work-to-life interrelation, (6) support, (7) working environment, (8) Big Five personality traits, (9) perceived meaningfulness and (10) sense of homeliness. Table 2 illustrates the allocation of the burnout factors to the, respectively, defined categories and their direction (+/–) in relation to their association with burnout.

**Table 2:**
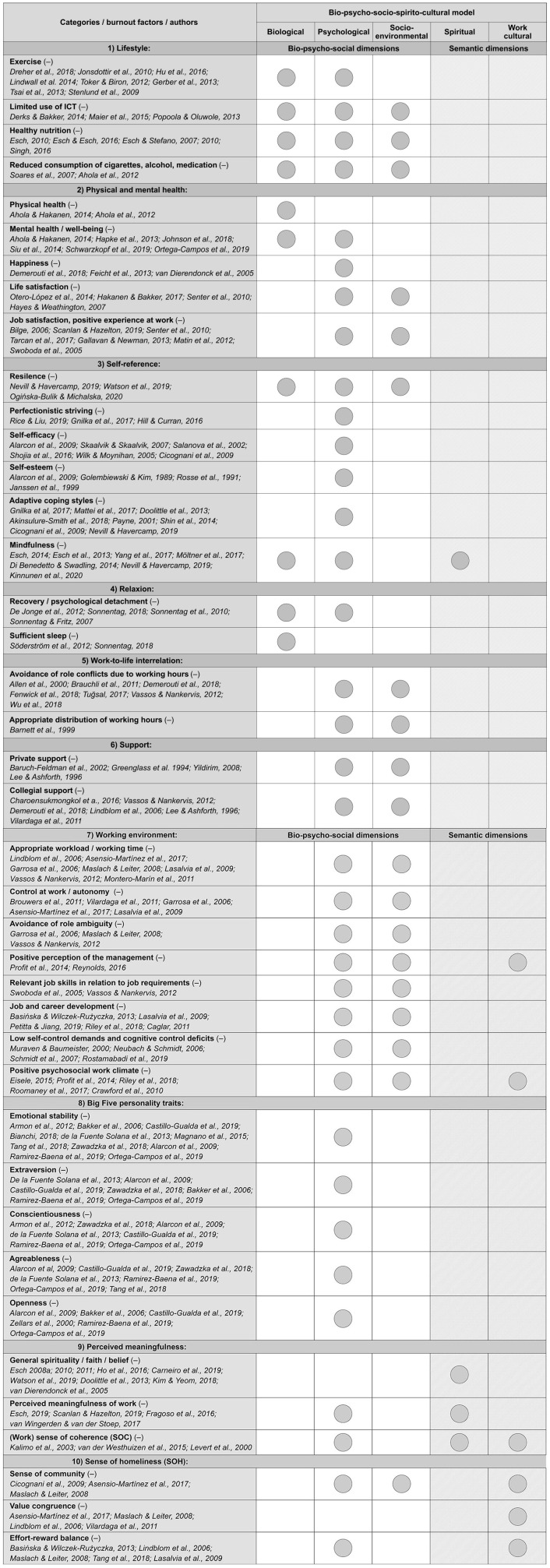
Identified burnout factors, their categories, direction of association with burnout and the assignment to the bio-psycho-socio-spirito-cultural model.

#### (1) Lifestyle

In the course of the literature search, various burnout factors were identified that can be assigned to lifestyle, such as *exercise, limited use of information and communication technology, healthy nutrition*, and *reduced consumption of cigarettes, alcohol, and medication*.

##### Exercise

Various studies prove the relation between exercise such as aerobic and anaerobic physical activities and its relationship with burnout (Jonsdottir et al., 2010; Toker and Biron, 2012; Lindwall et al., 2014; Hu et al., 2016; Dreher et al., 2018). According to Hu et al. (2016), workers who work more than 60 h a week and do not engage in any physical activity have the highest risk ratio of developing burnout. It was demonstrated that professionals who are engaged in light physical activity or moderate physical activity are less likely to report high levels of burnout and perceived stress compared to professionals who are not engaged in physical activity (sedentary lifestyle) (Stenlund et al., 2009; Jonsdottir et al., 2010; Gerber et al., 2013; Tsai et al., 2013; Dreher et al., 2018).

##### Limited Use of Information and Communication Technology (ICT)

ICT refers to the interaction with various devices (mobile phone, computer, etc.) and programs (e.g., applications) and can be associated with the development of technostress (stress that results from the use of technological devices) (Listopad and Brünner, 2020). Various identified studies demonstrated the relationship between the use of ICT and burnout (Popoola and Oluwole, 2013; Derks and Bakker, 2014; Maier et al., 2015). According to Popoola and Oluwole (2013) as well as Maier et al. (2015), the use of ICT can lead to technostress, which can contribute to techno-exhaustion, which in turn is significantly associated with work exhaustion and burnout. Their findings highlight that smartphone use is moderately positively correlated with emotional exhaustion and can prevent professionals from building psychological detachment to work-related issues which is important for relaxation, and which can help professionals to recover from stress (Sonnentag and Fritz, 2007; Sonnentag et al., 2010; De Jonge et al., 2012; Sonnentag, 2018). Another study reports that digitalization and the related technostress can be associated with technology-related work-home conflicts, invasion of privacy, work overload, avoidance of role ambiguity, and job insecurity, thus promoting burnout and stress (Maier et al., 2015).

##### Healthy Nutrition

This factor includes foods that are rich in complex carbohydrates, proteins, Vitamin B, Vitamin C, and Magnesium (e.g., Esch and Stefano, 2007, 2010; Esch, 2011; Esch and Esch, 2016; Singh, 2016). Furthermore, the enjoyment of food as well as mindful eating also plays a central role in stress management (Esch and Esch, 2016). Currently there are only first indications regarding the negative connection between healthy nutrition and (chronic) stress (e.g., Esch and Stefano, 2007; Esch, 2010; Esch and Esch, 2016; Singh, 2016).

##### Reduced Consumption of Cigarettes, Alcohol, and Medication

This factor includes a lifestyle with various components. A cross-sectional study by Soares et al. (2007) with 3,591 women identified a relationship between an increased burnout risk and the consumption of cigarettes and psychoactive as well as somatic medication. Their analysis also showed that medications are associated with high burnout—regardless of age, financial burden, illness, work demands, depression, and somatic complaints. In addition, Ahola et al. (2012) demonstrated that burnout is associated with high alcohol consumption.

#### (2) Physical and Mental Health

The factors *physical health, mental health/well-being, happiness, life satisfaction*, and *job satisfaction/positive experience at work* are assigned to the category physical and mental health.

##### Physical Health

This factor encompasses various physical states. There is initial evidence regarding an association between burnout and obesity, musculoskeletal disorders, cardiovascular disease, onset of coronary heart disease, and type 2 diabetes (Ahola et al., 2012; Ahola and Hakanen, 2014).

##### Mental Health/Well-Being

Similar to WHO (World Health Organization) (2018), the factors (subjective) well-being and mental health are considered analogously. Mental health is a state in which people can cope with daily stressors, work productively, and are able to contribute to the community [WHO (World Health Organization), 2018]. Well-being applies to inter- and intraindividual levels of positive functioning (e.g., relationships with others, self-referential attitudes, personal growth) (Burns, 2016). A significant relationship between burnout and depression, as well as between burnout and anxiety has been demonstrated by Koutsimani et al. (2019) and Ortega-Campos et al. (2019). Similar correlations between burnout and mental disorders, as well as burnout and well-being, are also shown by other authors (e.g., Hapke et al., 2013; Ahola and Hakanen, 2014; Siu et al., 2014; Johnson et al., 2018; Schwarzkopf et al., 2019).

##### Happiness

According to Diener (2000), the factor happiness can be defined as the moods and emotional reactions of workers to circumstances at work and at home, so that they play a central role—both inside and outside organizations (Seligman, 2002). Happiness thus refers to the affective and subjective evaluation of people's lives. People experience happiness when they feel many pleasant and few unpleasant emotions (Diener, 2000). Various authors demonstrate that happiness is negatively associated with burnout and stress (Van Dierendonck et al., 2005; Feicht et al., 2013). Moreover, Demerouti et al. (2018) showed that generally perceived happiness is not only strongly negatively associated with exhaustion, but also strongly positively associated with disengagement at work.

##### Life Satisfaction

Life satisfaction is a multi-factorial construct with affective-evaluative and cognitive-evaluative components. The affective components are characterized by the presence of positive emotions and the absence of negative ones. In contrast, the cognitive-evaluative components are composed of global and domain-specific satisfaction in various areas of life (Diener et al., 1985). The negative relationship between life satisfaction and burnout have been shown by various authors (e.g., Hayes and Weathington, 2007; Senter et al., 2010; Otero-López et al., 2014; Hakanen and Bakker, 2017).

##### Job Satisfaction/Positive Experience at Work

This factor includes the personal attitude of an employee toward work. It can also be linked to a sense of competence and success at work (Gallavan and Newman, 2013). According to various studies, low job satisfaction in different occupations and industries is associated with significantly higher levels of burnout (Senter et al., 2010; Matin et al., 2012; Scanlan and Hazelton, 2019), while increased job satisfaction or positive experience at work are negatively related to burnout and corresponding subscales (Swoboda et al., 2005; Gallavan and Newman, 2013; Tarcan et al., 2017; Scanlan and Hazelton, 2019). Likewise, a study by Bilge (2006) discovered that the most important variable that predicts all three Maslach's burnout subscales is intrinsic job satisfaction.

#### (3) Self-Reference

The factors *resilience, perfectionistic striving, self-efficacy, self-esteem, adaptive coping styles*, and *mindfulness* identified in the literature can be assigned to the category self-reference.

##### Resilience

This factor encompasses the ability to cope with stressful life situations using personal and socially mediated resources, and to maintain health (Fletcher and Sarkar, 2013; Färber and Rosendahl, 2018). Resilience is not exclusively a psychological phenomenon. Besides cognitive behavior, it is also assigned to the area of relaxation and mindfulness, as well as physical training and sports (Stahl et al., 2015; Esch, 2020). In the current review, it is assigned to self-reference. Besides health, resilience is related to factors such as coping style and optimism (Gillespie et al., 2007; Windle, 2010). Nevill and Havercamp (2019) showed weak negative correlations between resilience and emotional exhaustion, as well as resilience and depersonalization, while there was a weak positive association between resilience and professional efficacy. Other studies support the negative relationship between resilience and burnout (Watson et al., 2019; Ogińska-Bulik and Michalska, 2021).

##### Perfectionistic Striving

Perfectionistic striving describes the employees' intention to do things perfectly (Rice and Liu, 2019). According to various identified studies perfectionistic striving is negatively related to burnout (Hill and Curran, 2016; Gnilka et al., 2017; Rice and Liu, 2019). In a meta-analysis, Rice and Liu (2019) found a weak negative relationship between perfectionist striving and burnout, reduced personal efficiency, and depersonalization, and no significant relationship between perfectionist striving and emotional exhaustion.

##### Self-Efficacy

Self-efficacy means that people have the belief that they have some control over their actions and therefore trust in being able to achieve a certain goal (Bandura, 1997). Recent studies suggest that self-efficacy is negatively related to burnout (e.g., Salanova et al., 2002; Alarcon et al., 2009; Cicognani et al., 2009; Shojia et al., 2016). In a study by Skaalvik and Skaalvik (2007), self-efficacy correlates negatively with emotional exhaustion, depersonalization, and reduced professional efficacy. This association is also supported by Wilk and Moynihan (2005), who demonstrated that self-efficacy is negatively associated with emotional exhaustion.

##### Self-Esteem

This factor is generally defined as a global self-assessment (Alarcon et al., 2009), which consists of two aspects; belief in one's own abilities and belief in one's basic value (Locke et al., 1996). Based on recent literature, there is evidence that self-esteem is negatively related to burnout and subscales such as emotional exhaustion, depersonalization, and personal accomplishment (Rosse et al., 1991; Janssen et al., 1999; Alarcon et al., 2009), whereas low self-esteem is positively related to burnout (Golembiewski and Kim, 1989).

##### Adaptive Coping Styles

Coping strategies are generally considered to occur at the cognitive and behavioral level (Werdecker and Esch, 2018). In modern stress theory, biological and psychological stress mechanisms (McEwen, 2007; Werdecker and Esch, 2018) are combined according to the concept of the allostatic load (“stability through change”) by (Sterling and Eyer, 1988, p. 636), which is an extension of the concept of homeostasis (Selye, 1973; Esch, 2003). Adaptive coping styles comprises various functional coping strategies in dealing with stress. The negative relation between adaptive coping styles (e.g., positive reframing, using emotional support, humor) and burnout or (chronic) stress has been confirmed by various studies and within various professional groups (Cicognani et al., 2009; Doolittle et al., 2013; Shin et al., 2014; Gnilka et al., 2017; Mattei et al., 2017). Furthermore, there is also evidence that a positive relationship between dysfunctional coping strategies (e.g., denial, behavioral disengagement, self-blame) and burnout exists (Payne, 2001; Cicognani et al., 2009; Doolittle et al., 2013; Shin et al., 2014; Mattei et al., 2017; Akinsulure-Smith et al., 2018; Nevill and Havercamp, 2019). Consequently, the need for adaptive coping strategies is important for individuals in dealing with stress and burnout.

##### Mindfulness

Mindfulness can be described as the ability to focus attention on the current moment in a non-judgmental manner (Kabat-Zinn, 1991). A high level of mindfulness is associated with a lower level of burnout and chronic stress (Esch et al., 2013; Di Benedetto and Swadling, 2014; Esch, 2014; Yang et al., 2017; Nevill and Havercamp, 2019). Other identified studies have shown that mindfulness training, contemplation, and meditation are negatively associated with burnout and chronic stress (Möltner et al., 2017; Kinnunen et al., 2020).

#### (4) Relaxation

The factors *recovery/psychological detachment* and *sufficient sleep* are assigned to category relaxation in the present review.

##### Recovery/Psychological Detachment

Recovery activities and psychological detachment are closely connected. Recovery activities refer to what people do after work (e.g., physical activity, reading books) and thereby experience psychological detachment from work (can be differentiated into cognitive, emotional, and physical detachment) (De Jonge et al., 2012; Niks et al., 2017). Various authors identified a positive relationship between few recovery activities (and resulting low psychological detachment) and emotional exhaustion, psychosomatic complaints, disengagement, or even creativity at work (Sonnentag and Fritz, 2007; Sonnentag et al., 2010; Hakanen and Bakker, 2017; Sonnentag, 2018). Furthermore, De Jonge et al. (2012) showed a negative relationship between emotional as well as physical detachment and emotional exhaustion. According to Sonnentag et al. (2010), a high workload, emotional dissonance, and low work spatial boundaries at home (e.g., through home office) are associated with poor psychological detachment from work during non-working time, while a poor psychological detachment from work is positively associated with emotional exhaustion, low work engagement, psychosomatic complaints, and the need for recovery.

##### Sufficient Sleep

This factor refers to a sleep duration of more than 6 h, as has been defined by Söderström et al. (2012). In a 2-year longitudinal study, Söderström et al. (2012) identified insufficient sleep as the main risk for burnout among employees from different industries. Sleep has a central role in coping with demands. It has been shown that people can be more sensitive to emotional and stressful stimuli and events during sleep deprivation (Vandekerckhove and Cluydts, 2010; Gothe et al., 2019). An increase in burnout or burnout-related symptoms such as physical fatigue, reduced performance, and emotional exhaustion can occur more frequently with reduced sleep (Söderström et al., 2012; Sonnentag, 2018).

#### (5) Work-to-Life Interrelation

Two burnout-related factors that we identified in the literature can be assigned to the category work-to-life interrelation: *avoidance of role conflicts due to working hours* and *appropriate distribution of working hours*.

##### Avoidance of Role Conflicts Due to Working Hours

This factor means the prevention of difficulties in balancing work and private life and associated role conflicts (e.g., mother vs. manager). Various studies identified that conflicts between work and private life are positively related to burnout in various occupational groups and industries (e.g., Allen et al., 2000; Brauchli et al., 2011; Vassos and Nankervis, 2012; Demerouti et al., 2018; Fenwick et al., 2018; Wu et al., 2018). Similarly, emotional exhaustion decreases with increasing work-life accordance (Tugsal, 2017). The time-related role conflicts between work and private life do not only relate to the family, but also to conflicts outside the family such as leisure time and engagement (friends, volunteer work, etc.).

##### Appropriate Distribution of Working Hours

This factor represents the need for appropriate distribution of working hours among family members. Employees, whose working hours are more or less than they and their partner would like, tend to be more distant, distracted, and alienated at work than colleagues who are satisfied with their working hours. The same applies to employees whose working hours are distributed differently than they and their partner would prefer (Barnett et al., 1999). Even if employees have voluntarily reduced their working hours, the association with burnout at work is less strong the more their own working hours and those of their partner are perceived as appropriate. The relationship between the number of working hours and burnout thus depends on the extent to which the working hours meet the requirements of the employee, their partner and possibly their children (Barnett et al., 1999).

#### (6) Support

The factors *private support* and *collegial support* are assigned to the category support.

##### Private Support

This factor describes a variety of social sources in dealing with work-related stress. A study with traffic police officers showed that private support (e.g., family, friends) is negatively associated with burnout and positively associated with job satisfaction and productivity (Baruch-Feldman et al., 2002). Other studies also demonstrated the relationship between family support and burnout (Greenglass et al., 1994; Lee and Ashforth, 1996; Yildirim, 2008).

##### Collegial Support

It includes support from individuals within the organization. Various studies demonstrated a positive association between lack of support by superiors or coworkers and one or more aspects of burnout (Vilardaga et al., 2011; Vassos and Nankervis, 2012); a negative relationship was found for the burnout subscale professional efficacy (Lindblom et al., 2006). Similarly, other authors found a negative relationship between supervisor/coworker support and burnout (Lee and Ashforth, 1996; Charoensukmongkol et al., 2016; Demerouti et al., 2018), while Charoensukmongkol et al. (2016) found coworker support was negatively associated with emotional exhaustion and depersonalization.

#### (7) Working Environment

The burnout factors *appropriate workload*/*working time, control at work/autonomy, avoidance of role ambiguity, positive perception of the management, relevant job skills in relation to job requirements, job and career development, low self-control demands and cognitive control deficits*, and *positive psychosocial work climate* identified in the current literature can be assigned to the category working environment.

##### Appropriate Workload/Working Time

This factor represents the demands placed on employees during their work, which may be a high workload that is associated with overtime. According to various studies, a high workload and long working hours are associated with increased burnout (Garrosa et al., 2006; Lindblom et al., 2006; Maslach and Leiter, 2008, 2016; Vassos and Nankervis, 2012), while a low or manageable workload seems to be a protective factor against the risk of burnout (Lasalvia et al., 2009; Asensio-Martínez et al., 2017). For example, in a study of university employees, Montero-Marín et al. (2011) showed that people who work more than 40 h a week have a higher risk of burnout than people who work <35 h a week.

##### Control at Work/Autonomy

Control at work or autonomy means to be able to influence both conditions and activities at the workplace (Frese and Zapf, 1994). Various authors have demonstrated that low work control at work/low autonomy is associated with an increased risk of burnout (Garrosa et al., 2006; Lasalvia et al., 2009; Brouwers et al., 2011; Vilardaga et al., 2011; Asensio-Martínez et al., 2017).

##### Avoidance of Role Ambiguity

In the current review, role ambiguity describes the lack of a clear allocation of responsibilities for the work being carried out (Maslach and Leiter, 2008). Various identified studies highlight the positive relationship between role ambiguity and burnout (Garrosa et al., 2006; Maslach and Leiter, 2008; Vassos and Nankervis, 2012).

##### Positive Perception of the Management

This factor describes the employees' individual perception of management (i.e., the feeling of guidance and leadership, interpersonal job demands) in the company. Profit et al. (2014) demonstrated a negative association between positive perception of the management and emotional exhaustion, while Reynolds (2016) found that a negative perception of management was associated with increased burnout.

##### Relevant Job Skills in Relation to Job Requirements

This factor means the avoidance of professional overload. This implies that the existing job skills/resources of employees are sufficient to cope with the content-related-demands at work. According to Vassos and Nankervis (2012), there is a significant positive moderate relationship between lack of resources and emotional exhaustion, while no significant relationship to depersonalization and personal accomplishment could be identified. Furthermore, according to Swoboda et al. (2005), a lack of job skills in relation to job requirements is a risk factor for burnout.

##### Job and Career Development

This factor consists of various facets. These facets represent the development within the organization (consisting of little uncertainty and career development) and to organizational confidence with respect to general trust in the principal, colleague, and client (Caglar, 2011). According to different authors, job insecurity and lack of career development are associated with burnout (Lasalvia et al., 2009; Basińska and Wilczek-Ruzyczka, 2013; Riley et al., 2018; Petitta and Jiang, 2019). In addition, organizational confidence was identified as an aspect that is related to burnout (Caglar, 2011).

##### Low Self-Control Demands and Cognitive Control Deficits

This factor describes the control and inhibition of undesired behavior and emotions such as anger at work (e.g., in the service sector). Self-control occurs when people try to change the way they would spontaneously feel, think, or behave, which is associated with stress and resource consumption (e.g., through physiological arousal) (Muraven and Baumeister, 2000; Neubach and Schmidt, 2006; Schmidt et al., 2007). According to Schmidt et al. (2007), employees tend to rate their work as more strenuous in terms of perceived self-control demands if they are prone to severe cognitive control deficits. Thus, the level of self-control demands might reflect not only the objective work situation but also, to a lesser extent, the (in-)ability to control oneself. The self-control demands are correlated moderately positively with emotional exhaustion and depersonalization, while cognitive control deficits are strongly positively correlated with the two burnout subscales (Schmidt et al., 2007). This finding is also consistent with the job demand-control model (JDC) by Karasek (1979). In the JDC model, psychological demands and control are defined as key aspects of work in relation to the development of burnout and stress (Rostamabadi et al., 2019).

##### Positive Psychosocial Work Climate

This factor describes the psychological work climate between employees in an organization. According to Roomaney et al. (2017), interpersonal conflict at work is a significant predictor for emotional exhaustion and depersonalization. Other authors also found associations between psychosocial work climate and burnout or stress (Crawford et al., 2010; Profit et al., 2014; Eisele, 2015; Riley et al., 2018).

#### (8) Big Five Personality Traits

The factors *emotional stability, extraversion, conscientiousness, agreeableness*, and *openness* are assigned to the category of Big Five personality traits.

##### Emotional Stability

Emotional stability is the general tendency to be free of negative emotions such as frustration, irritability, guilt, as well as depression and anxiety; the opposite is called neuroticism (Costa and McCrae, 1992; McCrae and John, 1992; Dinkel, 2008). Various studies have shown a positive correlation between neuroticism and burnout for different professions and industries (Bakker et al., 2006; Alarcon et al., 2009; Armon et al., 2012; Magnano et al., 2015; Bianchi, 2018; Tang et al., 2018; Castillo-Gualda et al., 2019). Specifically, neuroticism is positively associated with emotional exhaustion, depersonalization, and reduced professional efficacy (Zawadzka et al., 2018), while there is a negative association between neuroticism and professional efficacy (De la Fuente Solana et al., 2013; Ortega-Campos et al., 2019; Ramirez-Baena et al., 2019).

##### Extraversion

Extraversion is the extent to which one is enthusiastic, active, sociable, and fun-loving (Costa and McCrae, 1992; McCrae and John, 1992; Dinkel, 2008). According to various authors, a high level of extraversion is associated with two or more aspects of burnout (Bakker et al., 2006; Castillo-Gualda et al., 2019). The personality trait is negatively correlated with emotional exhaustion, depersonalization, and reduced professional efficacy (Zawadzka et al., 2018), whereas the relationship between extraversion and professional efficacy is positive (Alarcon et al., 2009; De la Fuente Solana et al., 2013; Ortega-Campos et al., 2019; Ramirez-Baena et al., 2019).

##### Conscientiousness

This personality trait reflects the extent to which one is well-organized, self-disciplined, planful, achievement oriented, and responsible (Costa and McCrae, 1992; McCrae and John, 1992; Dinkel, 2008; Alarcon et al., 2009). Studies demonstrated a negative relationship between conscientiousness and burnout (Armon et al., 2012; Castillo-Gualda et al., 2019). Furthermore, several studies identified a negative correlation between conscientiousness and emotional exhaustion, depersonalization, as well as reduced professional efficacy (Zawadzka et al., 2018; Ortega-Campos et al., 2019), while a positive relationship was found between conscientiousness and professional efficacy (Alarcon et al., 2009; De la Fuente Solana et al., 2013; Ramirez-Baena et al., 2019).

##### Agreeableness

Agreeableness is the extent to which someone is cooperative, trusting, and sympathetic toward others (Costa and McCrae, 1992). Individuals with a high value in this personality trait tend to describe themselves as cooperative, altruistic, and sensitive (Magnano et al., 2015). The negative association between agreeableness and burnout has been demonstrated in various studies (Bakker et al., 2006; Tang et al., 2018). Similarly, other authors demonstrated negative associations between agreeableness and emotional exhaustion, agreeableness and depersonalization, as well as agreeableness and reduced professional efficacy (Zawadzka et al., 2018), whereas the relationship between agreeableness and professional efficacy was positive (Alarcon et al., 2009; De la Fuente Solana et al., 2013; Castillo-Gualda et al., 2019; Ortega-Campos et al., 2019; Ramirez-Baena et al., 2019).

##### Openness

Openness is the extent one is open to culture and experience (Magnano et al., 2015). In the identified literature, this personality trait shows a weak, and repeatedly non-significant correlation with burnout or corresponding subscales (Zellars et al., 2000; Bakker et al., 2006; Alarcon et al., 2009; De la Fuente Solana et al., 2013; Castillo-Gualda et al., 2019; Ramirez-Baena et al., 2019). According to various authors, openness is negatively associated with emotional exhaustion and depersonalization and positively associated with professional efficacy (Castillo-Gualda et al., 2019; Ortega-Campos et al., 2019).

#### (9) Perceived Meaningfulness

*General spirituality/faith/belief, perceived meaningfulness of work*, and *(work) sense of coherence* are burnout factors that we have identified in the current literature and assigned to category perceived meaningfulness.

##### General Spirituality/Faith/Belief

This factor refers to a feeling or belief of being connected with something “higher.” Something that will still be there before and after oneself and that is beyond everyday experience, e.g., God or nature (Esch, 2011). The relationship between spirituality, faith, belief and burnout or stress was examined in several general population studies (Van Dierendonck et al., 2005; Esch, 2008a,b, 2010, 2011, 2017, 2019; Doolittle et al., 2013; Daniel, 2014; Ho et al., 2016; Yang et al., 2017; Watson et al., 2019). Initial evidence points to the direction that individuals who are more religious or spiritual than others tend to possess higher resilience levels and are therefore less likely to be affected by burnout (Carneiro et al., 2019). Various studies support the negative relationship between spirituality and burnout (Ho et al., 2016; Kim and Yeom, 2018). In addition, there is a negative relationship between spirituality and emotional exhaustion, spirituality and depersonalization, and a positive relationship between spirituality and professional efficacy (Doolittle et al., 2013). Moreover, spiritual experiences and practices such as prayer, meditation, contemplation, mindfulness training, especially through the induction of reward mechanisms and the physiological relaxation response, can reduce pain and stress and thus promote health (e.g., Esch and Stefano, 2007, 2010; Esch, 2008a, 2011).

##### Perceived Meaningfulness of Work

This factor describes the individually perceived meaningfulness of work that exists independently of the perception of society (e.g., perception that one's own work has a socially assigned meaning) (Listopad et al., 2021). It is also associated with a sense of coherence (what happens on the outside of the individual resonates with what is felt inside) (Esch, 2019). Various authors identified that a greater sense of perceived meaningfulness of work is negatively related to burnout and positively related to work engagement (e.g., Fragoso et al., 2016; Van Wingerden and van der Stoep, 2017; Van Wingerden et al., 2018; Esch, 2019; Scanlan and Hazelton, 2019), which is why burnout can also be called as a “crisis of meaning” (Esch, 2019, p. 61). A study by Van Wingerden and van der Stoep (2017) found a strong negative correlation between perceived meaningful work and emotional exhaustion and between perceived meaningful work and cynicism, while the correlation between perceived meaningful work and work engagement was strongly positive. Another study (Listopad et al., 2021) investigating burnout based on an extended health and disease model, published after our database search, demonstrated a negative association between positive meaning and burnout, as well as meaning making through work and burnout, in the context of perceived meaningfulness of work. Similarly, positive meaning and work engagement, and meaning making through work and work engagement were positively associated. Since the publication of this study was outside the considered publication date, it is not included in the summary table. Nevertheless, these results highlight the relevance of perceived meaningfulness of work in relation with the syndrome.

##### (Work) Sense of Coherence (SOC)

This factor is composed of the elements sense of comprehensibility, manageability, and meaningfulness and is related to the salutogenesis (Antonovsky, 1987, 1996) to explore and strengthen health protection facets and resistance resources (Esch, 2020). A number of studies have transferred these three elements to the level of work and organization in order to provide a broadly applicable indicator of health-related working conditions together with work-related coherence. In this context, Bauer et al. (2015) found associations between work SOC and specific work-related stressors (e.g., time pressure, work interruptions) and resources (e.g., appreciation, holistic understanding about one's own work) as well as negative (exhaustion, psychosomatic complaints, etc.) and positive health outcomes (affective commitment, work engagement, etc.). A significant relationship between SOC and burnout or underlying subscales has been shown by two groups of authors in a cross-sectional and longitudinal study (Levert et al., 2000; Kalimo et al., 2003). It has also been demonstrated that the correlation between SOC and emotional exhaustion is stronger among employees between 51 and 60 years compared to younger age categories (Van der Westhuizen et al., 2015). A longitudinal study over ten years demonstrated that the SOC is a protective factor in relation to burnout (Kalimo et al., 2003).

#### (10) Sense of Homeliness

In the course of the literature search, various factors related to burnout were identified that can be assigned to a new overarching category sense of homeliness. The factors we assign to this category are *sense of community, value congruence*, and *effort-reward balance*.

##### Sense of Community

The literature search provides various factors that can be attributed to sense of community. Depending on the underlying concept, the factor sense of community encompasses aspects such as needs fulfillment, group membership, influence, emotional connection (McMillan and Chavis, 1986; Prezza et al., 1999), but also reward, fairness, values (Leiter and Maslach, 2006), communication, autonomy, corporate responsibility, etc. (Majer and D'Amato, 2001). A study by Cicognani et al. (2009) demonstrated a negative association between sense of community and burnout. Furthermore, according to Asensio-Martínez et al. (2017) and Maslach and Leiter (2008), there is a negative relationship between sense of community and emotional exhaustion, between sense of community and cynicism, as well as a positive relationship between sense of community and professional efficacy. A recent publication (Listopad et al., 2021) also investigated the subscales of sense of community. The authors identified a significant negative association between needs fulfillment and burnout, group membership and burnout, and emotional connection and burnout. The associations between needs fulfillment, group membership, and emotional connection and the dependent variable work engagement, in contrast, were positive (Listopad et al., 2021).

##### Value Congruence

This factor refers to the cognitive-emotional component of perceived congruence of professional goals, expectations, and personal value. It is thus closely linked to personal ideals and motivations that originally attracted employees to their work (Maslach and Leiter, 2008). According to the studies identified, there is a negative relationship between value congruence and burnout (Maslach and Leiter, 2008; Asensio-Martínez et al., 2017), while there is a positive association between disagreeing about values and burnout (Lindblom et al., 2006). Furthermore, the associations between low values commitment and emotional exhaustion, low values commitment and depersonalization, as well as low values commitment and reduced professional efficacy were positive (Vilardaga et al., 2011).

##### Effort-Reward Balance

The effort-reward imbalance (ERI) model is a model for explaining the development of stress in the work context. Siegrist (2002) assumes that gratification crises occur when the balance between work input and corresponding reward is not balanced from the employee's perspective. ERI can be measured with the ERI questionnaire (Siegrist et al., 2004) and includes the subscales effort, reward, and excessive commitment. We also assign to this factor the feeling of being treated appropriately by colleagues and leaders (subjective justice). Various authors found a positive relationship between ERI and burnout, and a negative association between subjective justice and burnout (e.g., Lindblom et al., 2006; Maslach and Leiter, 2008; Lasalvia et al., 2009; Tang et al., 2018). In this context, Basińska and Wilczek-Ruzyczka (2013) demonstrated various associations. For example, demands and lack of respect were positively associated with emotional exhaustion. Increased demands, lack of respect, and greater job security were positively associated with depersonalization. In contrast, higher demands and greater job security were positively associated with lower personal accomplishment.

### Socio-Demographic Factors

Some of the investigated studies point to relationships between socio-demographic factors such as work experience, gender, relationship status, having children or educational level and burnout. However, these findings are rather inconsistent so that we did not include them in the table of main findings. Despite, we provide a short overview of existing results in the following.

Although some studies suggest that young professionals with little work experience are more likely to develop burnout (e.g., Garrosa et al., 2006; Soares et al., 2007; De la Fuente Solana et al., 2013; Boštjančič et al., 2015; Simionato and Simpson, 2018), some of the identified studies show no significant relationship between age or work experience and burnout (e.g., Bilge, 2006; Yildirim, 2008; Matin et al., 2012; Tarcan et al., 2017; Ezenwaji et al., 2018). It is not possible to conclusively evaluate whether gender is related to burnout as some studies report no association (Yildirim, 2008; Matin et al., 2012; De la Fuente Solana et al., 2013; Ezenwaji et al., 2018). There is, however, isolated evidence regarding the relationship between gender and the burnout subscales (emotional exhaustions, depersonalization, and reduced professional efficacy). While men predominantly tend toward higher levels of depersonalization (Bilge, 2006; Vassos and Nankervis, 2012; Cañadas-De la Fuente et al., 2015), an increased value for emotional exhaustion has been reported among women (Frese and Zapf, 1994). In terms of marital status, according to Cañadas-De la Fuente et al. (2018), there is an association between single or divorced individuals and burnout (especially for men). Matin et al. (2012), on the other hand, could not identify any associations between marital status and burnout. Thus, the results regarding the connection between the relationship status and burnout are also inconclusive (e.g., Maslach et al., 2001; Maslach, 2003; Bekker et al., 2005; Lin et al., 2009; Al-Turki, 2010; Matin et al., 2012; Ayala and Carnero, 2013; De la Fuente Solana et al., 2013; Cañadas-De la Fuente et al., 2015, 2018). Furthermore, the results for having children are also inconsistent, as in some studies, childlessness is positively associated with burnout (Qu and Wang, 2015; Cañadas-De la Fuente et al., 2018) and in others, there are no associations (Coffey and Coleman, 2001; Queiros et al., 2013). The relationship between the level of education and burnout is equally inconclusive. While, according to Soares et al. (2007), a poorer level of education is correlated with an increased risk of burnout, Maslach et al. (2001) showed that people with a higher level of education tend to have a higher degree of burnout. The missing relationship between education and burnout is also supported by the study of Matin et al. (2012).

### Assignment of Factors to the Model of Health and Disease (Research Question 3)

The bio-psycho-social model of health and disease was postulated more than four decades ago by Engel (1977). According to the model, biological, psychological, and socio-environmental aspects play an important role in the development of health and disease and should therefore be considered in the description, prevention, and treatment of diseases (Engel, 1977; Egger, 2008; Havelka et al., 2009; Babalola et al., 2017; Lehman et al., 2017). In general, the (i) *biological* dimension refers to the physical elements of the body that influence and determine mental and physical health (Havelka et al., 2009; Lehman et al., 2017). The (ii) *psychological* dimension consists of cognitive, emotional, motivational, attitudinal, and behavioral aspects and encompasses the role of self, identity, personality, as well as various coping strategies (Suls and Rothman, 2004; Egger, 2008; Lehman et al., 2017). The (iii) *socio-environmental* dimension refers to the physico-chemical aspects such as air pollutants, nitrogen oxides, work environment, workplace conditions, but also to interpersonal dynamics of the work or family environment (Egger, 2008; Lehman et al., 2017). To answer our third research question of whether the bio-psycho-social model is sufficient to comprehensively describe the pathogenesis of burnout, we assigned each of the identified factors to the dimensions of the health and disease model. Many of the factors can unambiguously be assigned to one or more of these dimensions. However, as various authors postulated (Salmoirago-Blotcher et al., 2016; Saad et al., 2017; Esch, 2019; Listopad et al., 2021), several factors that have been identified to be related to burnout cannot easily be attributed to biological, psychological, and socio-environmental dimensions. Instead, they refer to spirituality and work culture. Hence, we add two (sub-)dimensions in line with propositions by Esch (e.g., 2008a, 2010, 2019) and Listopad et al. (2021), namely a (iv) *spiritual* and a (v) *work cultural* dimension. According to Esch (2008a, 2010, 2019) and Listopad et al. (2021), spirituality and work culture (as semantic dimensions) also play an important role in stress management and the development of burnout. The dimension spirituality refers to implicit/subjective factors (i.e., psychological-mental aspects such as inner/intrapersonal resources) and includes various spiritual experiences and practices such as prayer, meditation, contemplation, mindfulness, and perceived meaningfulness (Esch, 2008a, 2010, 2011, 2017, 2019). Work culture—as a further dimension—is described as an “inner home” (Esch, 2017, p. 149), i.e., subjective connectedness, belongingness, or sense of community (consisting of needs fulfillment, group membership, influence, and emotional connection) and represents a coherence between the outer world (e.g., values of the employer), and the inner world (e.g., own values) (Listopad et al., 2021). In the following, we describe the attribution of the factors to the model dimensions.

#### (1) Lifestyle

We assign the factors *exercise, limited use of ICT, healthy nutrition*, and *reduced consumption of cigarettes, alcohol, and medication* to the (i) biological dimension due to neurobiological processes involved. Furthermore, we also assign *exercise, limited use of ICT, healthy nutrition*, and *reduced consumption of cigarettes, alcohol, and medication* to the (ii) psychological dimension, as it represents individual aspects of attitude and behavior and can be attributed to, e.g., maladaptive coping strategies. The factors *limited use of ICT, healthy nutrition*, and *reduced consumption of cigarettes, alcohol, and medication* are also allocated to the (iii) socio-environmental dimension, due to their physico-chemical character.

#### (2) Physical and Mental Health

In this category, we assign the factors *physical health* and *mental health/well-being* to the (i) biological dimension, e.g., due to metabolic changes in the brain, neurotransmitters involved such as serotonin, dopamine, and norepinephrine, as well as genetic strain (Blows, 2000; Dfarhud et al., 2014; Woolfson, 2019). Furthermore, we assign *mental health/well-being, happiness, life satisfaction*, and *job satisfaction/positive experience at work* to the (ii) psychological dimension through its relationship to cognitive, emotional, motivational, attitudinal, and behavioral aspects. We also allocate *life satisfaction* and *job satisfaction/positive experience at work* to the (iii) *socio-environmental dimension* due to the interpersonal dynamics in private and professional life and their relation to satisfaction.

#### (3) Self-Reference

We allocate the burnout factors *resilience* and *mindfulness* to the (i) biological dimension as it can also be linked with, e.g., relaxation, mindfulness, physical training, and sports (Esch, 2020). In addition, we assign *resilience, perfectionistic striving, self-efficacy, self-esteem, adaptive coping styles, and mindfulness* to the (ii) psychological dimension, based on individual attitude and behavioral aspects, as well as the role of self and identity. Beyond that, we assign *resilience* to the (iii) socio-environmental dimension due to resilience-promoting aspects such as family, friends, and work. In the last step within this category, we assign *mindfulness* to the (iv) spiritual dimension, since it frequently represents a spiritual practice (Esch and Stefano, 2004; Esch, 2011).

#### (4) Relaxation

We assign the factors *recovery/psychological detachment* and *sufficient sleep* to the (i) biological dimension due to sleep physiology, neurobiological processes, and the effects of the factors with the body (Van Dongen et al., 2003; Sanford et al., 2015). In addition, we assign *recovery/psychological detachment* to the (ii) psychological dimension, as it is associated with motivational, attitudinal, and behavioral aspects of individuals and can also be a part of a coping strategy (Sonnentag and Fritz, 2007; Lehman et al., 2017).

#### (5) Work-to-Life Interrelation

The factors *avoidance of role conflicts due to working hours* and *appropriate distribution of working hours* are assigned to the (ii) psychological dimension due to the emotional, attitude, and behavioral aspects within the health and disease model. Moreover, we assign these two factors to the (iii) socio-environmental dimension because of the corresponding interpersonal dynamics within the family, friends, etc.

#### (6) Support

Both factors, i.e., *private support* and *collegial support* are assigned to the (ii) psychological dimension, since it represents individual coping strategies (Sonnentag and Fritz, 2007). Similarly, we also assign these factors to the (iii) socio-environmental dimension because, e.g., there are interpersonal dynamics (e.g., family, friends) and social support is an external factor within the work environment.

#### (7) Working Environment

We assign all factors, i.e., *appropriate workload/working time, control at work/autonomy, avoidance of role ambiguity, positive perception of the management, relevant job skills in relation to job requirements, job and career development, low self-control demands and cognitive control deficits*, as well as *positive psychosocial work climate* to the (ii) psychological dimension due to the cognitive, emotional, attitudinal, and behavioral aspects. We also assign all mentioned factors to the (3) socio-environmental dimension due to the interpersonal dynamics of the working environment (e.g., among co-workers, supervisors) as well as immediate work environment and working conditions. *Positive perception of the management* and *positive psychosocial work climate* are assigned to the (v) work cultural dimension, as the perceived connectedness and belongingness could be disturbed.

#### (8) Big Five Personality Traits

All five factors, i.e., *emotional stability, extraversion, conscientiousness, agreeableness*, and *openness* are assigned to the (ii) psychological dimension, since they are associated with the personality and the identity of a person.

#### (9) Perceived Meaningfulness

We assign *perceived meaningfulness of work* and *(work) SOC* to the (ii) psychological dimension, as it comprises cognitive, emotional, motivational, attitudinal, and behavioral aspects and can encompasses the role of self, identity, and personality. Furthermore, we assign various burnout factors from this category to the new semantic dimensions (spirituality and work culture). This means that we assign the factors *general spirituality/faith/belief*, *perceived meaningfulness of work*, and *(work) SOC* to the (iv) spiritual dimension, as the factors refer to psychological-mental aspects and includes various spiritual experiences and practices such as prayer, meditation, contemplation, or meaningfulness (Esch, 2008a, 2010, 2011, 2017, 2019). Similarly, a prior study (Listopad et al., 2021) assigned perceived meaningfulness to the spiritual dimension within an extended model of health and disease. We finally assign *(work) SOC* to the (v) work cultural dimension through the subjective belongingness and the corresponding sense of coherence between the outer world (e.g., work) as well as the inner world (e.g., personal meaningfulness, personal goals).

#### (10) Sense of Homeliness

We allocate *sense of community* and *effort-reward balance* to the (ii) psychological dimension because these factors can affect emotion, cognition, attitude, and motivation on an individual level and could impact experience and behavior. We also assign *sense of community* to the (iii) socio-environmental dimension through the interpersonal dynamics within an organization or team. Finally, the factors *sense of community, value congruence*, and *effort-reward balance* are allocated to the (v) work cultural dimension due to the feeling of being connected to a company. Furthermore, the assignment of the sense of community factor to the work cultural dimension is also supported by a recently published empirical study (Listopad et al., 2021).

**Figure 2:**
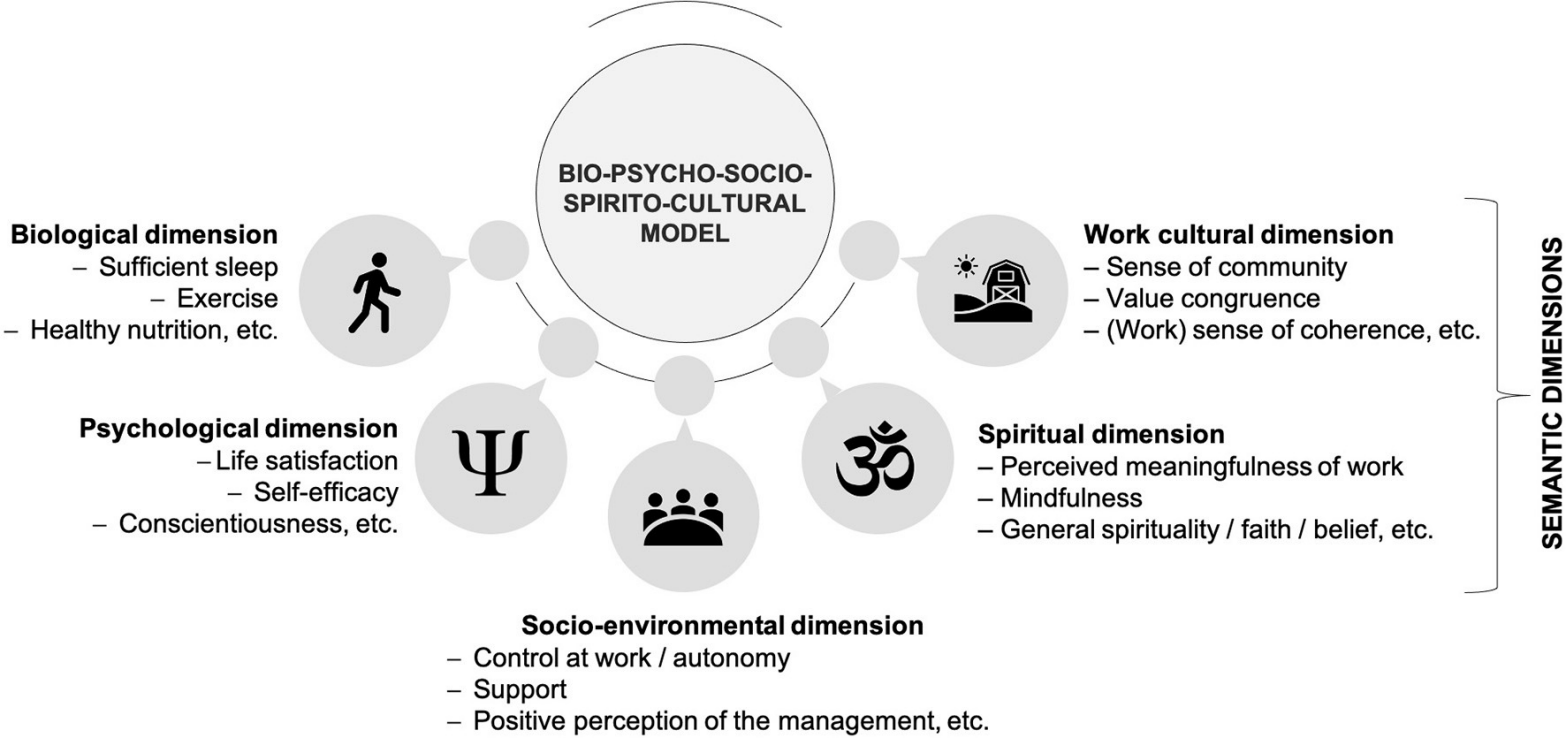
The bio-psycho-socio-spirito-cultural model (extension of the existing model).

Based on the discussed literature, we conclude that the currently used bio-psycho-social model is not sufficient to describe the pathogenesis of burnout, since not all identified burnout factors can be assigned to the three dimensions of the health and disease model. Both a spiritual and a work cultural dimension (semantic dimensions) are needed to adequately consider all factors related to burnout. Consequently, from a health care perspective, the description of burnout should therefore be broadened to prevent evasive diagnoses [such as “occupational phenomenon;” Esch, 2019; Listopad et al., 2021; WHO (World Health Organization), 2021] and to describe burnout as a disease. Figure 2 illustrates the extended model based on burnout. It is pointed out that the burnout factors can also be assigned to several dimensions simultaneously.

## Discussion

This is the first review to discuss the onset of burnout based on an extended model of health and disease. In the following, we present a critical summary of the main findings, report limitations and directions for future research, discuss the relevance for an extended health and disease model, and present practical applications.

In total, we have identified 40 factors related to burnout. Among the analyzed studies, burnout has been measured using various instruments (e.g., MBI, Maslach et al., 1996; OLBI, Demerouti et al., 2001; SMBM, Shirom and Melamed, 2006) that differentiate subscales of burnout to varying degrees, making it difficult to interpret, distinguish, and compare the corresponding burnout subscales. Consequently, the comparability and interpretation of the results (e.g., in terms of strength and subscales) in the present study may be limited. For these reasons, future studies should consider a differentiated analysis between the various measuring instruments, corresponding subscales, and the respective correlations of different factors. Future research should also attempt to prioritize burnout factors by strength, for example, through meta-analytic approaches.

Beyond that, we have defined 10 overarching categories from the identified burnout factors. Since the assignment of factors to the categories was the result of a reconciliation process that was performed by all authors, in the future, a larger study with many experts in the field could help to find a larger consensus on the assignment of factors to categories, e.g., using the Delphi method.

We started by allocating the identified burnout factors to the dimensions of the health and disease model (Engel, 1977). Since the biological, psychological, and socio-environmental dimensions were not sufficient to describe the onset of burnout, we extended the model by additional semantic dimensions (consisting of a spiritual dimension and work cultural dimension). However, the exact relationship between the burnout factors and the corresponding biological, psychological, socio-environmental, spiritual, and work cultural dimensions require further investigation. Future empirical studies could examine the relationships in a differentiated and more extensive way (i.e., consideration of age, education level, industry, occupation, all burnout subscales). It would also be interesting to examine whether the semantic dimensions can also be applied to the development of other diseases (e.g., depression, personality disorders, post-traumatic stress disorder).

### Research Limitations and Recommendations for Future Studies

A shortcoming of the present study is that it has focused on burnout factors among working adults. Burnout will be attributed to work factors in the ICD-11, where it is not defined as a diagnosis. Instead, in the ICD-11, it is currently described outside the extended health and disease model. Based on the results, we propose the application and extension of the bio-psycho-social model in the context of burnout. Moreover, in this context, it is still necessary to investigate how individuals outside of classical wage labor (e.g., students, homemaker) are affected by burnout.

The other shortcoming within this review is the undifferentiated treatment of the results from various professions and industries. Future research should consider a greater differentiation of factors associated with burnout regarding professions and industries.

Another point that needs to be addressed concerns the term “work cultural.” In this review, the term is solely applied to the work context and should not be interpreted otherwise. This means that the diverse cultural aspects of the respective populations in the selected studies (e.g., Europe compared to Asia) have not been considered. For example, work and spirituality/religion may have different personal and social importance in various cultures and can, therefore, be associated with health and illness (also burnout) in distinct ways. Future investigations may take this aspect into account.

In addition, there is still an overlap between burnout and depression in research and practice (e.g., measuring burnout with depression inventories, or diagnosis of depression even though burnout is present) (Bianchi et al., 2015). In this context, days of absence due to diagnosed depression (but in the presence of burnout symptoms) should be critically discussed in further investigations, as causes, treatment, and prevention may differ (Koutsimani et al., 2019; Listopad et al., 2021). Future studies should examine burnout and depression in greater detail by differential diagnosis.

We have identified studies (e.g., Esch and Stefano, 2007, 2010; Esch, 2010; Esch and Esch, 2016; Singh, 2016) that demonstrate the association between nutrition and (chronic) stress, but no additional study on the association between nutrition and burnout, which should be further investigated in the future.

Finally, our systematic narrative review has limitations due to the methodological approach. Based on the identified burnout factors, future empirical studies could use the positive deviance approach to examine individuals who do not suffer from burnout in the same work environment.

### Relevance of the Extended Health and Disease Model for Burnout

It could be shown that the current model of health and disease is not sufficient to explain the pathogenesis of burnout, since the identified factors can be assigned not only to the (i) biological, (ii) psychological, and (iii) socio-environmental dimension, but also to further (iv) spiritual and (v) work cultural *semantic* dimensions, as has previously been postulated by Esch (2008a, 2011, 2019) and Listopad et al. (2021). The health care system perspective should therefore find a new and appropriate approach to burnout that could expand the understanding of the “phenomenon”—or even the disease. In this way, burnout could be adequately diagnosed and treated in those affected.

As long as burnout is described outside the extended health and disease model, it might lead to evasive diagnoses (Esch, 2019; Listopad et al., 2021) such as “occupational phenomenon” [WHO (World Health Organization), 2021]. In this context, subsuming the various factors under the term “chronic workplace stress” [WHO (World Health Organization), 2021] is insufficient and of limited use for research and practice (e.g., in the treatment of affected people, prevention courses). Consequently, burnout definitions should be based on the extended health and disease model in the future development of ICD-12 (Listopad et al., 2021).

### Practical Applications

Various identified burnout factors can be prevented (positive relationship) or strengthened (negative relationship), e.g., through group courses that promote health. There are various (multimodal) health-promotion programs such as the BERN (**B**ehavior, **E**xercise, **R**elaxation, and **N**utrition) stress management concept (Esch and Stefano, 2007, 2010; Esch, 2011), MBSR (Mindfulness-Based Stress Reduction), MBCT (Mindfulness-Based Cognitive Therapy), meditation (e.g., Zen and mantra meditation), breathing techniques, Yoga, or Qigong (Michaelsen et al., 2021). Thus, it would still have to be evaluated whether the already known health-promoting measures strengthen the right resources (also considering the identified factors assigned to spirituality and work culture). At this point, we also refer to research on the effectiveness of programs in promoting resources that can be assigned to semantic dimensions (e.g., perceived meaningfulness of work, [work] SOC, sense of community, value congruence).

## Conclusion

The factors associated with burnout are numerous and the relationships between these factors are complex. Nevertheless, the present review has examined a wide range of studies to obtain an up-to-date understanding of factors that are related to burnout, which in turn point the way forward for further cross-sectional and longitudinal studies. In this context, we highlighted several research gaps in the study of burnout that require further investigation.

Overall, based on the studies reviewed, the bio-psycho-social model does not appear to be sufficient to assign all identified burnout factors to the established health and disease model. Thus, we provide evidence that an extension of the health and disease model is necessary to achieve an expanded understanding of burnout, to recognize it as a disease, and to adequately prevent and treat it. As long as burnout is not described within the extended model, it is not adequately captured.

In addition, the review highlights that the three subscales of the burnout concept according to Maslach et al. (1996) may not be sufficient, as it was not developed based on the extended health and disease model. Consequently, the concept of burnout would have to be investigated and possibly extended based on a bio-psycho-socio-spirito-cultural model.

## Data Availability Statement

The original contributions presented in the study are included in the article, further inquiries can be directed to the corresponding author.

## Author Contributions

IL was responsible for initial searches, article screening, data extraction, interpretation of the data, as well as writing and critical revision of the manuscript. MM and TE provided support through the conception and design of the study and contributed to the final manuscript by making corrections within each section. LW supported through critical reading and feedback of the manuscript. All authors approved the final manuscript.

## Conflict of Interest

The authors declare that the research was conducted in the absence of any commercial or financial relationships that could be construed as a potential conflict of interest.

## Publisher's Note

All claims expressed in this article are solely those of the authors and do not necessarily represent those of their affiliated organizations, or those of the publisher, the editors and the reviewers. Any product that may be evaluated in this article, or claim that may be made by its manufacturer, is not guaranteed or endorsed by the publisher.

## Footnotes

^1^ICD-11 is the eleventh International edition of the Classification of Diseases.

^2^The literature search in PSYNDEX for publication references of German psychological has been neglected, as the literature search in PubPsych already covers it.

^3^The search in the PsycINFO database was not used as the recommended minimum number of databases was reached. According to Green et al. (2006), it is recommended to use at least two databases for the literature research in a narrative review, so that the prerequisite corresponds to current literature recommendations.
